# Continuous Electric Furnace Annealing as a Sustainable Route: Influence on the Microstructure, Texture, and Mechanical Properties of Cold-Rolled Low-Carbon Steels for CO_2_ Emission Reduction

**DOI:** 10.3390/ma19081626

**Published:** 2026-04-17

**Authors:** Sanjaya Kumar Pradhan, Young-Gon Kim, Inn-Hyup Jeong, Yu-Seong Lee, Youn-Ja Kim, Kyeong-Cheol Baek, Sung-Min Joo, Min-Suk Oh

**Affiliations:** 1 Division of Advanced Materials Engineering, Department of Energy Storage/Conversion Engineering of Graduate School, Jeonbuk National University, Jeonju 54896, Republic of Korea; sanjayap@jbnu.ac.kr (S.K.P.); innhyup17@naver.com (I.-H.J.);; 2 Purpose Built Mobility Group, Korea Institute of Industrial Technology (KITECH), Gwangju 61012, Republic of Korea; 3 R&D Department, SAMWOOECO Co., Ltd., Gwangyang 57759, Republic of Korea; 4 Department of Naval Architecture and Ocean Engineering, Chosun University, Gwangju 61452, Republic of Korea

**Keywords:** continuous electric furnace, annealing, low-carbon steel, texture, CO_2_ emission

## Abstract

Steel processing requires energy-efficient heat-treatment routes without compromising material performance. Traditional annealing furnaces used for low-carbon (LC) steels are energy-intensive and major contributors to CO_2_ emissions, creating a need for sustainable alternatives. This study evaluates continuous electric furnace (CEF) annealing as a low-emission route to tailor the microstructure, texture, and mechanical properties of cold-rolled LC steel. Samples were annealed at 750 °C and 850 °C for 60 s, followed by comprehensive microstructural and crystallographic characterization using XRD, SEM, EBSD (IPF, GOS, KAM, ODF), hardness, and tensile testing. Annealing increased recrystallization from ~4% in the as-rolled condition to ~98% at 850 °C, reduced the mean KAM from 1.9° to 0.1°, enhanced the high-angle grain boundary fraction to 0.91, and promoted γ-fiber strengthening while suppressing detrimental θ-fiber components. The 850 °C condition achieved optimal mechanical performance (UTS × TE = 11.1 GPa%). These results demonstrate that CEF annealing enables sustainable processing with better mechanical performance in LC steels.

## 1. Introduction

Electrification of heat-treatment operations in the steel industry is increasingly recognized as a key lever for deep decarbonization while maintaining or improving product quality [1]. The iron and steel sector currently accounts for approximately 7–9% of global energy-related CO_2_ emissions, making it one of the most emission-intensive industrial sectors worldwide [2,3,4,5]. In conventional processing routes, a substantial fraction of these emissions arises from fossil fuel combustion in reheating and annealing furnaces, where natural gas, coke oven gas, or oil are burned to generate high-temperature atmospheres for strip and sheet processing [6,7,8]. As climate policies become more stringent, regions such as the European Union, East Asia, and North America are urging steel producers to align with net-zero or near-zero emission trajectories, in which direct fossil fuel use in process heating is progressively replaced by electricity or other low-carbon energy sources [2,9]. This transition has stimulated growing interest in electrified annealing of full-hard low-carbon (LC) steels, where multiple annealing steps along the processing route imply that even modest emission reductions per cycle can accumulate into substantial lifecycle benefits.

Within this broader decarbonization framework, commercial-quality LC steels used in automotive, construction, and general forming applications constitute a significant share of global steel strip production [10,11]. These steels are typically cold-rolled to high reductions and subsequently annealed to restore ductility, refine grain structure, and promote favorable γ-fiber recrystallization textures that enhance formability [12,13,14]. Extensive research has explored the microstructural evolution, texture development, recrystallization behavior, and mechanical properties of LC steels following cold rolling and annealing [15,16,17,18,19]. For example, Muljono, Ferry, and Dunne [20] systematically investigated the influence of heating rate on recrystallization behavior in low- and ultra-low-carbon steels, demonstrating that increasing heating rates shift the nose of the recrystallization curve and can refine grain size when combined with appropriate annealing temperatures. Complementary studies on ultra-rapid or flash annealing have shown that short-duration, high-temperature treatments can produce fully recrystallized microstructures with refined grains and optimized strength–ductility combinations in LC steels [21,22]. Recent work by Leão et al. [23] further emphasized that recrystallization kinetics must be carefully controlled to achieve target yield strength and ductility, particularly when integrating high-heating-rate routes into industrial processing lines.

Cai et al. [24] examined interstitial-free steels subjected to ultra-short annealing and reported a strong coupling between microstructural evolution and texture development. Ray, Jonas, and Hook [25] provided a comprehensive review of cold-rolling and annealing textures in low- and extra-low-carbon steels, showing that the cold-rolling texture is dominated by α-fiber (<110>//RD) and θ-fiber (<001>//ND) components, whereas annealing promotes the development of γ-fiber (<111>//ND) textures that are crucial for enhanced mechanical performance and formability. More recently, Aghamohammadi et al. [26] demonstrated that controlled post-annealing promotes recrystallization-driven texture transformation in deformed ferritic steel, leading to modified α/γ-fiber intensities and improved ductility through microstructural refinement. Similarly, Duan et al. [27] investigated texture evolution during annealing of a low-carbon formable strip steel containing impurity elements and showed that impurity additions can suppress the development of strong γ-fiber textures while increasing the fraction of random orientations.

Although prior studies have extensively explored the influence of cold rolling and annealing parameters on the microstructural and textural evolution, as well as the mechanical properties of LC steels, these investigations have predominantly employed conventional annealing furnaces, which are energy-intensive and significant contributors to CO_2_ emissions. Furthermore, most studies have varied annealing parameters such as time, temperature, and heating rate while holding the furnace type constant. In contrast, the potential impact of furnace electrification itself on microstructural evolution and texture development has received comparatively limited attention.

In this context, the primary objective of the present study is to investigate and optimize recrystallization behavior under CEF annealing conditions. Specifically, this work focuses on identifying the effective temperature window within a CEF required to achieve the desired microstructural evolution, texture development, and mechanical properties in cold-rolled low-carbon steel. The study is designed to provide a fundamental understanding of recrystallization kinetics under controlled electric furnace conditions, rather than to present a direct comparative experimental analysis with conventional furnaces. Additionally, the adoption of electric furnace processing offers the potential to reduce direct CO_2_ emissions, contributing to more sustainable steel processing routes.

## 2. Materials and Methods

### 2.1. Materials

Cold-rolled low-carbon steel sheets (1 mm thickness, POSCO Co., Ltd., Pohang, Republic of Korea) with a nominal composition of Fe–0.04C–0.25Mn–0.019P–0.015S (wt.%) were used in this study. The chemical composition of the steel was determined using optical emission spectroscopy. The sheets were subjected to CEF annealing at 650 °C, 750 °C, 800 °C and 850 °C for 60 s. The CEF employed in this study utilizes SiC heating elements, with steel sheet heating achieved predominantly via radiant transfer from these elements. The furnace facilitates rapid heating rates of ~80.3 °C/s to target annealing temperatures, enabling short processing times compatible with continuous annealing lines. Air was used as the process atmosphere in the CEF annealing. The annealing temperatures (650–850 °C) were selected based on the typical recrystallization range of cold-rolled low-carbon steels (~600–800 °C), enabling the assessment of partial to complete recrystallization under CEF conditions. Temperature monitoring and control were achieved using K-type thermocouples, infrared pyrometers, and a data logging system, ensuring uniform and stable thermal exposure through symmetric SiC heater configuration and closed-loop control. Following annealing, specimens were sectioned into dimensions of 200 mm × 70 mm × 1 mm for tensile testing and 10 mm × 10 mm × 1 mm for microstructural and crystallographic characterization.

### 2.2. Characterization Methods

Phase identification of the as-rolled and annealed samples was performed using X-ray diffraction (XRD; RINT-2000, Rigaku, Tokyo, Japan) with monochromatic Cu–Kα radiation (λ = 1.5406 Å) over a 2θ range of 40–130°, employing a step size of 0.02° and a scanning rate of 2° min^−1^. Microstructural observations were conducted using field-emission scanning electron microscopy (FE-SEM; SUPRA40VP, Carl Zeiss Microscopy GmbH, Jena, Germany). Specimens were mechanically grounded (400–2400 grit), fine polished with 1 μm diamond suspension, and etched using 10% nital solution. For crystallographic characterization, electron backscatter diffraction (EBSD; JIB-4601F, JEOL, Tokyo, Japan) was carried out after electro-polishing at 20 V for 20 s in a solution of 80 vol.% acetic acid and 20 vol.% perchloric acid. EBSD scans were acquired over an area of 150 μm × 90 μm with a step size of 0.50 μm and analyzed using EDAX TSL-OIM v7.1. Data cleanup included confidence index standardization and neighbor orientation correlation (5° misorientation threshold, minimum CI = 0.1). Low-angle grain boundaries (LAGBs) and high-angle grain boundaries (HAGBs) were defined as 2° ≤ θ < 15° and 15° ≤ θ < 62.5°, respectively. Vickers hardness tests were conducted under a 200 gf load with a 10 s dwell time (five measurements per condition). Room-temperature tensile tests were performed at a strain rate of 3 × 10^−3^ s^−1^ using a universal testing machine (INSTRON 8801MTL6258, Norwood, MA, USA) in accordance with ASTM E8/E8M-22 [28], with three specimens tested per condition to ensure reproducibility. Tensile specimens were machined with their longitudinal axis parallel to the rolling direction (RD) of the cold-rolled steel sheets.

## 3. Results

### 3.1. Microstructure and Recrystallization Behavior

**Figure 1:**
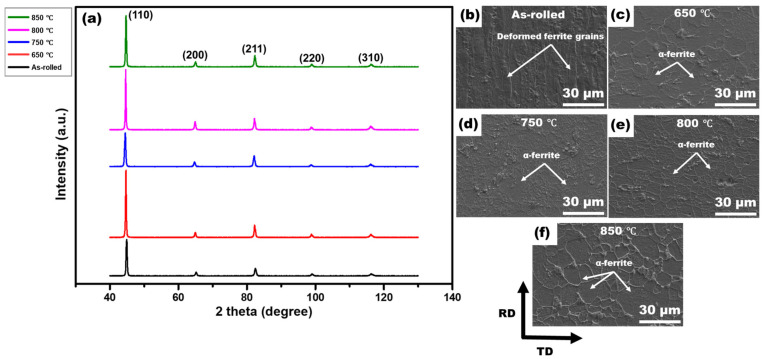
(**a**) XRD patterns and SEM micrographs of the LC steel in the as-rolled condition (**b**), and after annealing at 650 °C (**c**), 750 °C (**d**), 800 °C (**e**) and 850 °C (**f**). The diffraction peaks observed at (110), (200), (211), (220), and (310) were indexed according to the standard ICDD card No. 00-006-0696 for α-Fe (BCC ferrite).

Figure 1a shows the XRD patterns of as-rolled, 750–annealed, and 850–annealed LC steel samples. The BCC (α-ferrite) phase is detected in each condition with peaks at (110), (200), (211), (220), (310).

SEM micrographs of the as-rolled and annealed samples were obtained in the RD–transverse direction (TD) plane, as presented in Figure 1b–f. The as-rolled condition exhibits a heavily deformed microstructure characterized by elongated deformed ferrite grains aligned along the rolling direction. In contrast, after annealing at 650 °C and 750 °C, the microstructure shows the existence of newly formed equiaxed ferrite grains with a marked reduction in elongated deformation features, while the samples annealed at 800 °C and 850 °C display a fully equiaxed recrystallized ferritic microstructure with a uniform grain morphology.

**Figure 2:**
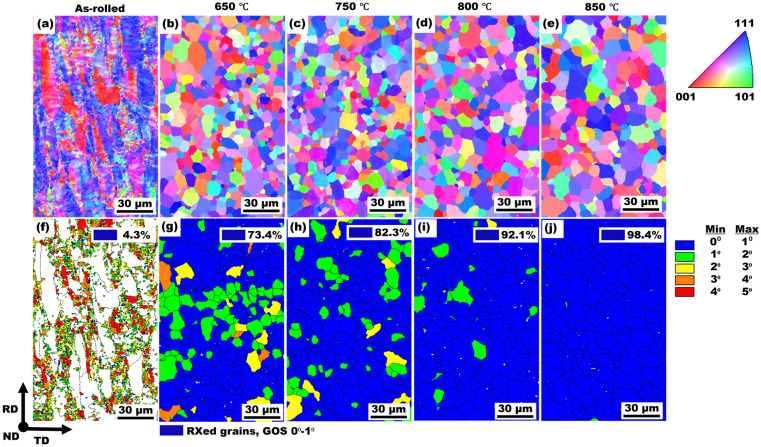
EBSD IPF maps (**a**–**e**) and corresponding grain orientation spread (GOS) maps (**f**–**j**) of the LC steel in the as-rolled condition (**a**,**f**) and after annealing at 650 °C (**b**,**g**), 750 °C (**c**,**h**), 800 °C (**d**,**i**), and 850 °C (**e**,**j**). RXed denotes recrystallized grains. (Online version in color).

EBSD inverse pole figure (IPF) maps and grain orientation spread (GOS) maps were acquired for the three processing conditions in the RD-TD plane, as shown in Figure 2.

IPF maps depict the crystallographic orientation of individual grains relative to the sample reference frame, while GOS maps quantitatively represent the average intragranular orientation deviation within each grain and are therefore used to distinguish deformed, recovered, and recrystallized grains based on stored lattice strain [29,30]. The IPF map of the as-rolled sample (Figure 2a) reveals that ferrite grains are predominantly aligned between <100> and <111> directions. The corresponding GOS map (Figure 2f) shows significantly high-GOS regions (GOS > 3°) across most grains, consistent with a largely unrecrystallized microstructure. Upon annealing at 650 °C and 750 °C, the IPF maps (Figure 2b,c) shows the development of equiaxed ferrite grains, respectively, together with a notable presence of grains exhibiting strong <111> orientation, although several grains are oriented along <110> and <100> directions. The associated GOS maps (Figure 2g,h) display a mixture of low- and high-GOS regions (GOS 0°–3°), corresponding to a recrystallized grain fraction of 73.4% and 82.3%, respectively. In the 800 °C and 850 °C annealed condition, the IPF maps (Figure 2d,e) are dominated by uniformly equiaxed grains with a crystallographic orientation like that observed after annealing at 650 °C and 750 °C. The GOS maps (Figure 2i,j) show predominantly low GOS values (GOS 0°–1°), indicating an almost fully recrystallized microstructure with a recrystallized fraction of 92.1% and 98.4%, respectively. 

**Figure 3:**
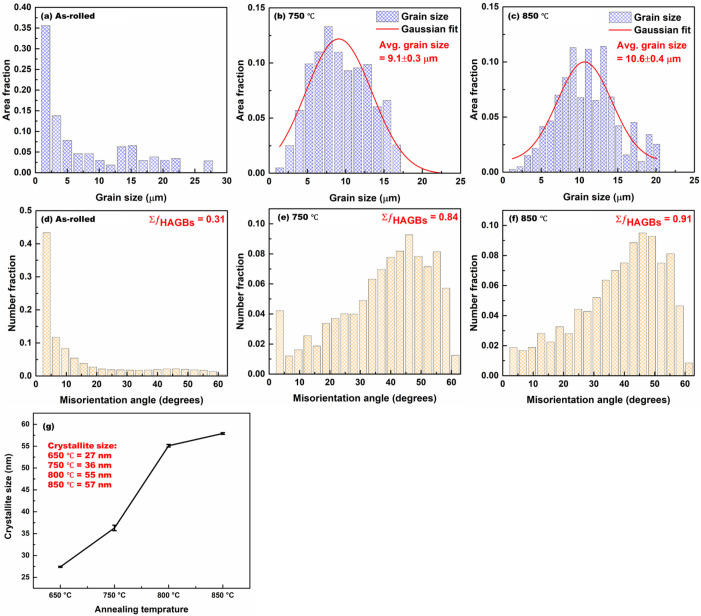
Grain size distributions (**a**–**c**) and grain boundary misorientation angle distributions (**d**–**f**) of the LC steel in the as-rolled condition (**a**,**d**) and after annealing at 750 °C (**b**,**e**) and 850 °C (**c**,**f**). Crystallite size was determined using Rietveld refinement, as presented in (**g**).

Figure 3 shows the grain size distributions and misorientation angle distributions for the as-rolled, 750–annealed, and 850–annealed samples.

The as-rolled condition exhibits a non-uniform grain size distribution, characteristic of heavy deformation. Annealing at 750 °C yields a uniform grain size distribution with an average grain size of 9.1 ± 0.3 µm. Further annealing at 850 °C produces a modest increase in average grain size (10.6 ± 0.4 µm). The misorientation angle distributions reveal a high fraction of LAGBs (0.69) in the as-rolled state, whereas annealed samples show a pronounced increase in the fraction of HAGBs, with the highest fraction (0.91) observed in the 850–annealed condition. Crystallite size was determined using Rietveld refinement, as presented in Figure 3g. The results show a decrease in crystallite size with increasing annealing temperature, consistent with the grain size distribution observed in Figure 3, providing further insight into the microstructural evolution.

**Figure 4:**
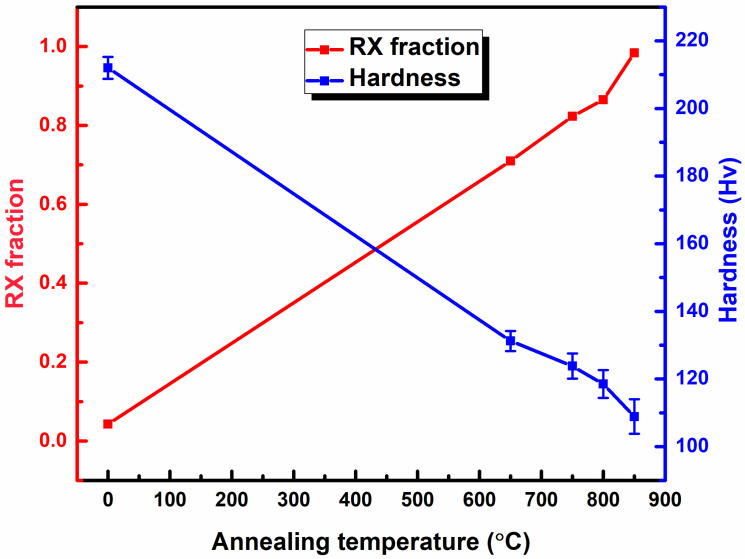
Quantified recrystallization fractions and corresponding Vickers hardness values of the as-rolled and annealed LC steel samples. RX denotes recrystallization and is used only in the axis labels and legends for clarity.

The measured recrystallization fractions, together with the corresponding Vickers hardness values for the as-rolled and annealed samples, are illustrated in Figure 4.

The recrystallization fraction increases systematically with annealing temperature, from nearly zero in the as-rolled state to a high fraction at 650 °C and 750 °C, and approaches full recrystallization after 800 °C. Conversely, the Vickers hardness decreases monotonically with increasing annealing temperature. The as-rolled sample exhibits the highest hardness, while the 850–annealed sample shows the lowest hardness, consistent with the progressive reduction in deformation structures.

**Figure 5:**
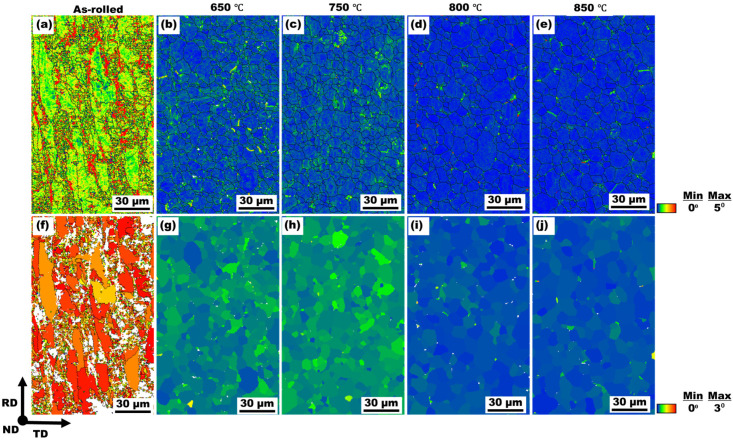
EBSD KAM maps (**a**–**e**) and corresponding GAM maps (**f**–**j**) of the LC steel in the as-rolled condition (**a**,**f**) and after annealing at 650 °C (**b**,**g**), 750 °C (**c**,**h**), 800 °C (**d**,**i**), and 850 °C (**e**,**j**). (Online version in color).

Figure 5a–e show EBSD kernel average misorientation (KAM) for the three conditions. The KAM map quantifies local lattice misorientation between neighboring pixels and thus provides a spatially resolved measure of local plastic strain within grains [30,31].

The as-rolled sample (Figure 5a) exhibits high KAM regions (yellow/red regions) distributed throughout the microstructure, reflecting a high density of lattice distortions. After annealing at 650 °C and 750 °C, the KAM maps (Figure 5b,c) show a mix of low and moderate KAM regions (blue to green), indicating a moderately recovered microstructure with some residual strain in certain grains. In the 800 °C and 850–annealed samples, the KAM maps (Figure 5d,e) show predominantly blue grains (low KAM regions), indicating extensive recovery of lattice strain. These trends are corroborated by the grain average misorientation (GAM) maps, as shown in Figure 5f–j, which measure the average misorientation of all pixels within an individual grain relative to its mean orientation and are therefore used as a quantitative indicator of the overall intragranular strain state [30,31]. The as-rolled sample (Figure 5f) exhibits high GAM regions (orange/red regions) distributed throughout the microstructure, reflecting a pronounced intragranular misorientation. In contrast, the 650 °C and 750–annealed sample (Figure 5g,h) shows a mix of low and high GAM regions (blue to green), consistent with a marked reduction in internal strain. Likewise, in the 800 °C and 850–annealed sample, the GAM maps (Figure 5i,j) show predominantly blue grains, indicating further reduction in intragranular strain.

**Figure 6:**
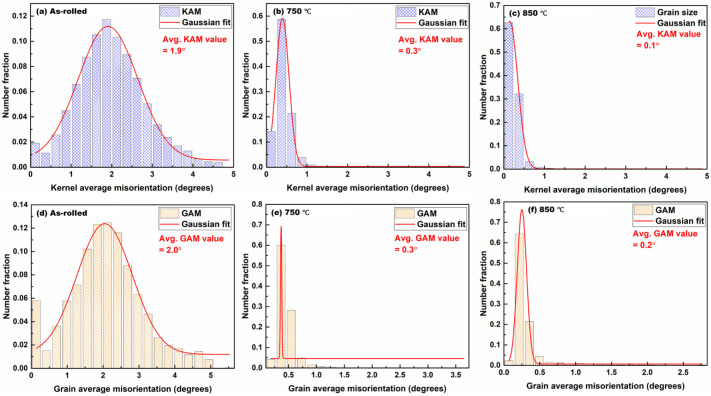
KAM distributions (**a**–**c**) and GAM distributions (**d**–**f**) of the LC steel in the as-rolled condition (**a**,**d**) and after annealing at 750 °C (**b**,**e**) and 850 °C (**c**,**f**).

Figure 6 presents the statistical distributions of KAM and GAM values for all samples. The as-rolled condition shows broad distributions with high weighted average KAM (1.9°) and GAM (2.0°) mean values, whereas annealing at 750 °C shifts the distributions toward lower misorientation values (KAM = 0.3°, GAM = 0.3°). The 850–annealed sample exhibits the narrowest distributions and the lowest average KAM (0.1°) and GAM (0.2°) values, reflecting a uniform and strain-free microstructure.

### 3.2. Microtextural Evolution

**Figure 7:**
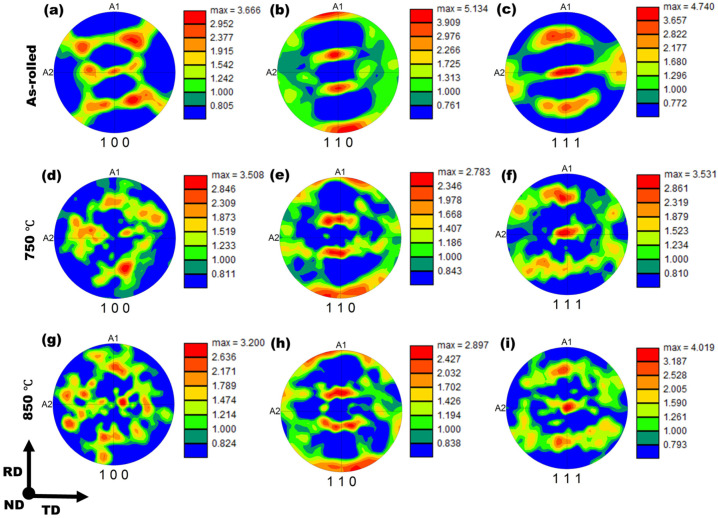
{100}, {110}, and {111} pole figures of the LC steel in the as-rolled condition (**a**–**c**) and after annealing at 750 °C (**d**–**f**) and 850 °C (**g**–**i**), illustrating the evolution of crystallographic texture with annealing temperature (online version in color).

To examine the effect of annealing on crystallographic texture evolution, the {100}, {110}, and {111} pole figures were derived from EBSD data for the as-rolled and annealed samples, as shown in Figure 7.

The pole figures of the 750 °C and 850 °C annealed samples exhibit textures that are distinctly different from the rolling-induced texture observed in the as-rolled condition. In all pole figures, the color scale denotes pole intensity as multiples of random distribution (MRD, or simply × R). The as-rolled sample displays comparatively higher MRD intensities, indicating a pronounced deformation texture. In particular, a strong {110} texture (Figure 7b) aligned with the rolling direction is observed, with a maximum MRD intensity of approximately 5.13, whereas the {100} and {111} components are comparatively weaker, with maximum intensities of ~3.66 × R and ~4.74 × R, respectively (Figure 7a,c). In contrast, both annealed samples show a relative enhancement of {100} and {111} texture intensities accompanied by a reduction in the intensity of the {110} component. Moreover, with increasing annealing temperature, the {111} texture becomes progressively stronger, reaching a maximum MRD intensity of ~4.01 in the 850 °C annealed sample (Figure 7i), compared to ~3.53R for the 750 °C sample (Figure 7f). Similar annealing-induced texture transitions have been reported for ferritic steels in the literature [32,33,34].

**Figure 8:**
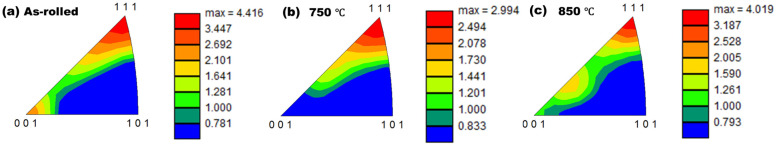
EBSD IPF texture maps of the LC steel in the as-rolled condition (**a**) and after annealing at 750 °C (**b**) and 850 °C (**c**), showing the evolution of crystallographic texture with annealing temperature (online version in color).

Moreover, the EBSD IPF texture maps of the as-rolled and annealed samples are acquired and shown in Figure 8.

The as-rolled sample exhibits crystallographic texture, characterized by dominant <100> and <111> orientations with maximum relative intensities of approximately 2.69 × R and 4.41 × R, respectively (Figure 8a). Upon annealing, a marked modification of the crystallographic texture is observed. The sample annealed at 750 °C displays a significantly weakened texture, exhibiting a maximum intensity of ~2.99 × R (Figure 8b). In contrast, the 850–annealed sample shows the development of a strong texture dominated by <111> orientations, reaching a maximum intensity of ~4.01 × R, accompanied by a comparatively weaker <112> component with an intensity of ~2.00 × R (Figure 8c).

**Figure 9:**
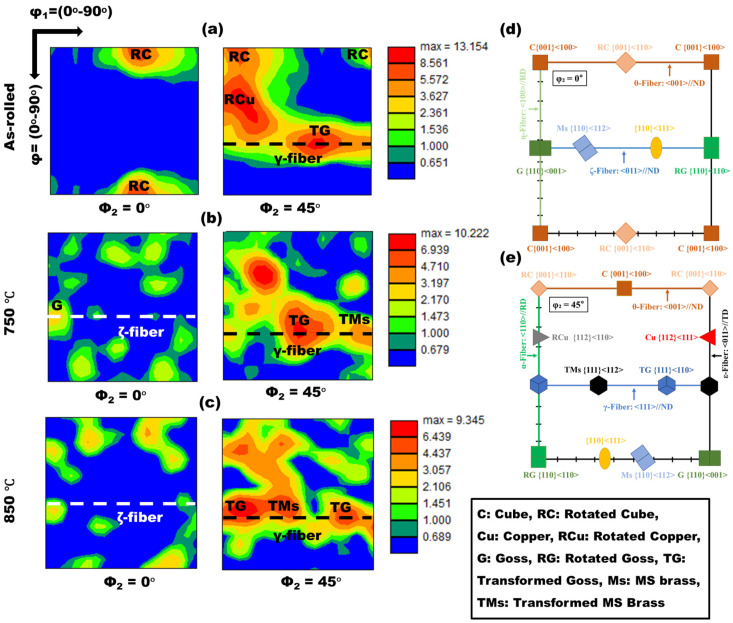
φ_2_ = 0° and 45° sections of the orientation distribution function (ODF) of the LC steel in the as-rolled condition (**a**) and after annealing at 750 °C (**b**) and 850 °C (**c**). (**d**,**e**) Schematic representation of the common crystallographic texture components in BCC polycrystalline materials, reproduced from Refs. [36,38]. (Online version in color).

Furthermore, the crystallographic texture components of the α-ferrite phase in the as-rolled and annealed samples are quantified using orientation distribution function (ODF) analysis. The ODF provides a quantitative framework for systematically characterizing and tracking the evolution of crystallographic texture components under different annealing conditions [35]. Figure 9a–c present the ODF plots of the ferrite phase for the respective conditions, calculated using Harmonic series expansion and displayed at φ_2_ = 0° and 45° sections with Bunge notations in Euler space. For reference, Figure 9d,e show the standard texture components of BCC ferrite at φ_2_ = 0° and 45°, respectively, facilitating systematic identification and comparison of the observed texture components. In the as-rolled condition (Figure 9a), a pronounced θ-fiber/Cube fiber texture (<001>//ND) is observed with an intensity of 5.57 × R. Within this fiber, the Rotated Cube (RC, {001}<110>) component exhibits a higher intensity of 7.06 × R.

The γ-fiber (<111>//ND) is strongly developed, showing an overall intensity of 8.56 × R, accompanied by prominent Transformed Goss (TG, {111}<110>) and Transformed MS Brass (TMs, {111}<112>) components with maximum intensities of 10.85 × R and 5.57 × R, respectively. In addition, a strong α-fiber texture (<110>//RD) is detected with an intensity of 8.56 × R, together with a pronounced Rotated Cu (RCu, {112}<110>) orientation exhibiting a peak intensity of 10.85 × R. Such texture characteristics, dominated by α- and γ-fiber components, are typical of cold-rolled BCC materials and have been widely reported in the literature [36,37]. Following annealing at 750 °C (Figure 9b), significant modification of the texture is observed. The Goss (G, {110}<001>) component appears on the ζ-fiber (<011>//ND) with an intensity of 3.19 × R, while the ζ-fiber itself exhibits a reduced intensity of 2.68 × R. The θ-fiber and α-fiber textures are markedly weakened to intensities of 2.17 × R and 3.19 × R, respectively, and the RC component on the θ-fiber is substantially reduced to 1.82 × R. Despite this overall weakening of rolling-related texture components, the γ-fiber remains comparatively well preserved, retaining an intensity of 6.93 × R, together with TG and TMs components showing intensities of 8.57 × R and 4.71 × R, respectively. With further annealing at 850 °C (Figure 9c), the ζ-fiber persists with an intensity of 2.57 × R, accompanied by the Goss component at 3.05 × R. The θ-fiber and α-fiber textures are further reduced to 2.10 × R and 3.05 × R, respectively. In contrast, the γ-fiber continues to exhibit pronounced intensity, reaching 5.43 × R, along with strong TG and TMs components displaying intensities of 7.88 × R and 6.43 × R, respectively. Overall, the ODF analysis reveals that post-rolling annealing leads to substantial redistribution of texture components, characterized by progressive weakening of deformation-induced θ- and α-fiber textures and the relative persistence and strengthening of γ-fiber-related orientations [36,38].

**Figure 10:**
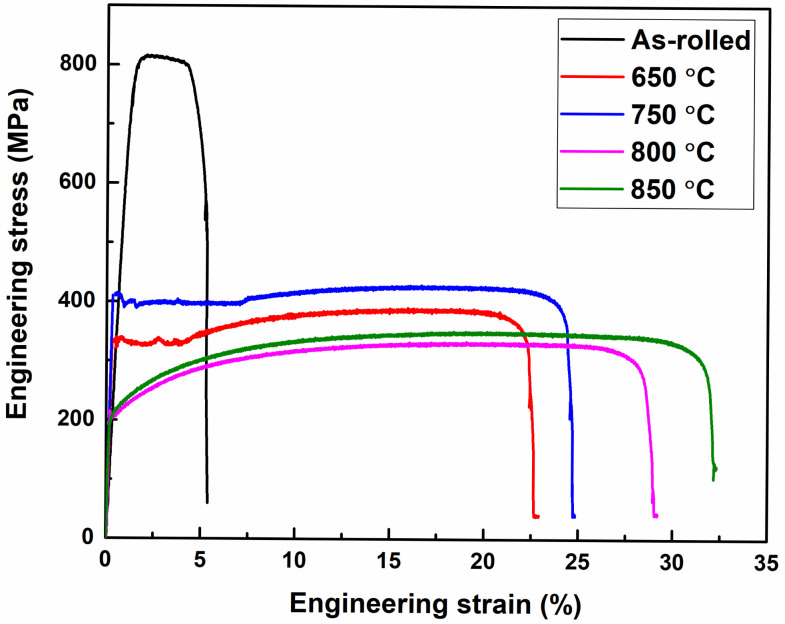
Engineering stress–strain curves for as-rolled and annealed samples.

Figure 10 presents the engineering stress–strain curves of the as-rolled and annealed samples. The as-rolled sample exhibits the highest ultimate tensile strength (UTS) but limited total tensile elongation (TE). Annealing at 650 °C and 750 °C results in a reduction in UTS, accompanied by a noticeable improvement in TE. Further annealing at 800 °C and 850 °C leads to the lowest strength level and the highest elongation among all conditions.

## 4. Discussion

The present study investigated CEF annealing as a sustainable approach to modify the microstructure, texture, and mechanical properties of cold-rolled LC steels. The systematic analysis of microstructural evolution through multiple characterization techniques provides critical insights into recrystallization behavior, textural development and their relationship with mechanical performance.

The transition from heavily deformed grains in the as-rolled condition to equiaxed microstructures following annealing demonstrates the effectiveness of short-duration CEF annealing at elevated temperatures. As confirmed by XRD and SEM analysis (Figure 1), the BCC α-ferrite phase remained predominant across all conditions, establishing a single-phase ferritic system characteristic of LC steels. The recrystallization fraction increased progressively from ~4% in the as-rolled state to ~82% at 750 °C and ~98% at 850 °C (Figure 2f–j), indicating temperature-dependent recrystallization kinetics. This observation aligns with classical recrystallization theory, where higher temperatures accelerate nucleation and growth rates through increased atomic mobility and reduced activation energy for dislocation annihilation [39]. The misorientation angle distributions revealed a marked increase in HAGBs with increasing annealing temperature, rising from 0.31 in the as-rolled state to 0.91 at 850 °C. This enhancement of HAGBs is significant, as these boundaries effectively impede crack propagation, contributing to improved ductility [40]. The combined effect of grain refinement (average grain size: 9.1 ± 0.3 μm at 750 °C and 10.6 ± 0.4 μm at 850 °C) and elevated HAGBs fractions explains the superior mechanical performance achieved in the annealed samples, with the PSE value increasing from 4.8 GPa.% in the as-rolled condition to 10.2–11.1 GPa.% in the annealed samples. KAM and GAM analysis (Figure 5) revealed a substantial reduction in internal strain with increasing annealing temperature. The mean KAM value decreased significantly from 1.9° in the as-rolled condition to 0.3° at 750 °C and further to 0.1° at 850 °C, indicating effective strain relaxation within the ferrite phase. This gradual relaxation of internal strain is attributed to annealing-induced recovery and recrystallization, which substantially lower the dislocation density and promote microstructural softening [41]. The associated decrease in Vickers hardness, from ~212 HV in the as-rolled state to ~123–108 HV after annealing, further corroborates that higher annealing temperatures enhance softening through progressive defect annihilation and recrystallization.

Texture analysis (Figure 7, Figure 8 and Figure 9) using pole figures, IPFs, and ODF sections demonstrated substantial redistribution of crystallographic orientations during recrystallization. The as-rolled deformation texture, characterized by pronounced α- and θ-fibers, underwent significant weakening at 750 °C and 850 °C. The presence of a pronounced θ-fiber component is known to adversely affect tensile performance; therefore, its intensity should be minimized to optimize mechanical properties [42]. Notably, the γ-fiber (⟨111⟩//ND) strengthened and shifted toward the ⟨111⟩ orientation, with maximum ODF intensity reaching 9.34 × R at 850 °C. The development of strong γ-fiber texture components is beneficial for formability and deep drawability in ferritic steels, as this orientation promotes favorable plastic strain distribution [43]. The persistence of well-defined γ-fiber components across both annealed conditions suggests selective growth mechanisms during recrystallization, wherein nuclei with preferred orientations exhibit enhanced growth rates into the deformed matrix [44,45,46]. Moreover, increasing the annealing temperature facilitates the activation of a larger number of potential nucleation sites, thereby enhancing the thermodynamic driving force and the overall probability of static recrystallization [47]. Consequently, the annealed samples develop newly formed grains with distinct and independently evolved crystallographic orientations.

The exceptional tensile elongation improvement (from 6% to 24–32%), coupled with reasonable strength retention (UTS: 425–349 MPa), in the annealed samples reflects the synergistic effect of microstructural refinement, HAGB enhancement, and favorable texture evolution. The 850–annealed sample, despite lower absolute strength, achieved the highest PSE value (11.1 GPa.%), indicating superior engineering relevance for applications requiring both formability and structural integrity. In contrast, the poor TE of approximately 6% observed in the as-rolled condition is associated with the predominance of θ-fiber-related texture components, including the detrimental Rotated Cube orientation, which are known to adversely affect ductility in ferritic steels [36]. Table 1 summarizes the tensile properties of the present CEF-annealed LC steels and compares them with those of traditionally annealed LC steels reported in previous studies.

This comprehensive microstructural modification through CEF annealing demonstrates its viability as a cost-effective, energy-efficient alternative to traditional annealing for achieving optimized mechanical properties in commercial LC steel.

## 5. Conclusions

CEF annealing was systematically evaluated as a sustainable route to tailor the microstructure, texture, and mechanical performance of cold-rolled LC steel. The principal conclusions are summarized as follows:

1. CEF annealing preserved the single-phase BCC α-ferrite while transforming heavily deformed elongated grains into equiaxed recrystallized grains. The recrystallization fraction increased from ~4% in the as-rolled condition to ~82% at 750 °C and ~98% at 850 °C.
2. Increasing annealing temperature promoted grain growth (9.1 ± 0.3 µm at 750 °C to 10.6 ± 0.4 µm at 850 °C) and significantly increased the fraction of HAGBs (from 0.31 to 0.91), indicating enhanced boundary mobility and recrystallization completion.
3. KAM and GAM analyses revealed substantial strain relaxation with increasing annealing temperature, with mean KAM values decreasing from 1.9° (as-rolled) to 0.3° (750 °C) and 0.1° (850 °C), accompanied by a pronounced reduction in hardness (from ~212 HV to ~108–123 HV), confirming effective recovery and recrystallization.
4. Texture evolution analysis demonstrated progressive weakening of deformation-induced α- and θ-fiber components and strengthening of the γ-fiber (<111>//ND) during annealing. The suppression of detrimental θ-fiber and cube-related orientations, together with enhanced γ-fiber intensity, contributed to improved plastic deformation behavior.
5. The tensile properties of the CEF-annealed steel samples are comparable to those reported for traditionally furnace-annealed LC steels, with total elongation of 24–32% and a PSE value of ~10–11 GPa·%, highlighting the effectiveness of CEF annealing in optimizing the microstructure–texture–tensile property relationship.

Collectively, the present results confirm that CEF annealing provides a technically viable and environmentally sustainable alternative to traditional furnace processing for full-hard LC steels, enabling microstructural refinement, favorable texture evolution, and superior mechanical performance while supporting decarbonization objectives.

## Figures and Tables

**Table 1:** Tensile properties of cold-rolled LC steel annealed in CEF and traditional furnaces.

Annealing Temperature (°C)	UTS (MPa)	TE (%)	PSE (GPa.%)	References
700	698	18	12.56	[26]
650	386	22	8.49	This study
750	425	24	10.20	This study
780	345	40	13.80	[24]
800	331	29	9.5	This study
850	405	24	9.72	[48]
850	349	32	11.16	This study
860	875	12	10.50	[49]
870	280	46	12.80	[22]
880	510	13	6.63	[50]

## Data Availability

The original contributions presented in this study are included in the article. Further inquiries can be directed to the corresponding authors.
